# Stochastic Population Dynamics of a Montane Ground-Dwelling Squirrel

**DOI:** 10.1371/journal.pone.0034379

**Published:** 2012-03-27

**Authors:** Jeffrey A. Hostetler, Eva Kneip, Dirk H. Van Vuren, Madan K. Oli

**Affiliations:** 1 Department of Wildlife Ecology and Conservation, University of Florida, Gainesville, Florida, United States of America; 2 Migratory Bird Center, Smithsonian Conservation Biology Institute, National Zoological Park, Washington, D.C., United States of America; 3 Department of Wildlife, Fish, and Conservation Biology, University of California Davis, Davis, California, United States of America; Australian Wildlife Conservancy, Australia

## Abstract

Understanding the causes and consequences of population fluctuations is a central goal of ecology. We used demographic data from a long-term (1990–2008) study and matrix population models to investigate factors and processes influencing the dynamics and persistence of a golden-mantled ground squirrel (*Callospermophilus lateralis*) population, inhabiting a dynamic subalpine habitat in Colorado, USA. The overall deterministic population growth rate *λ* was 0.94±SE 0.05 but it varied widely over time, ranging from 0.45±0.09 in 2006 to 1.50±0.12 in 2003, and was below replacement (*λ*<1) for 9 out of 18 years. The stochastic population growth rate *λ_s_* was 0.92, suggesting a declining population; however, the 95% CI on *λ_s_* included 1.0 (0.52–1.60). Stochastic elasticity analysis showed that survival of adult females, followed by survival of juvenile females and litter size, were potentially the most influential vital rates; analysis of life table response experiments revealed that the same three life history variables made the largest contributions to year-to year changes in *λ*. Population viability analysis revealed that, when the influences of density dependence and immigration were not considered, the population had a high (close to 1.0 in 50 years) probability of extinction. However, probability of extinction declined to as low as zero when density dependence and immigration were considered. Destabilizing effects of stochastic forces were counteracted by regulating effects of density dependence and rescue effects of immigration, which allowed our study population to bounce back from low densities and prevented extinction. These results suggest that dynamics and persistence of our study population are determined synergistically by density-dependence, stochastic forces, and immigration.

## Introduction

Understanding factors and processes that determine dynamics and persistence of biological populations is an important goal of ecology [1], [2], [3], [4]. Because many environments fluctuate stochastically, population dynamics of organisms inhabiting such environments are strongly influenced by unpredictable environmental variations. On the other hand, density dependent effects are presumed to be ubiquitous as well as an important force in regulating biological populations [4], [5], [6]. It is generally believed that both density-dependent (DD) and density-independent (DID) processes influence population dynamics, but the relative roles of DD regulation and DID destabilization are still debated [3], [7], [8], [9], [10]. The effects of DID processes on population dynamics are likely to become stronger due to global climate change, and it is critical to understand how stochastic variations and density-dependent mechanisms interact to influence population dynamics [7], [9].

Global climate change is predicted to impact both the mean and variance of climatic parameters and consequently, the mean and variance of demographic rates (e.g., survival and reproductive rates) [11], [12], [13], [14]. This can potentially exacerbate the effects of environmental variation on population demography as organisms are exposed to novel environmental conditions. Therefore, understanding the demographic effects of environmental variability is critical since these perturbations are likely to influence the long-term growth rate, persistence, and resilience of populations inhabiting variable environments [13], [15], [16], [17].

**Figure 1:**
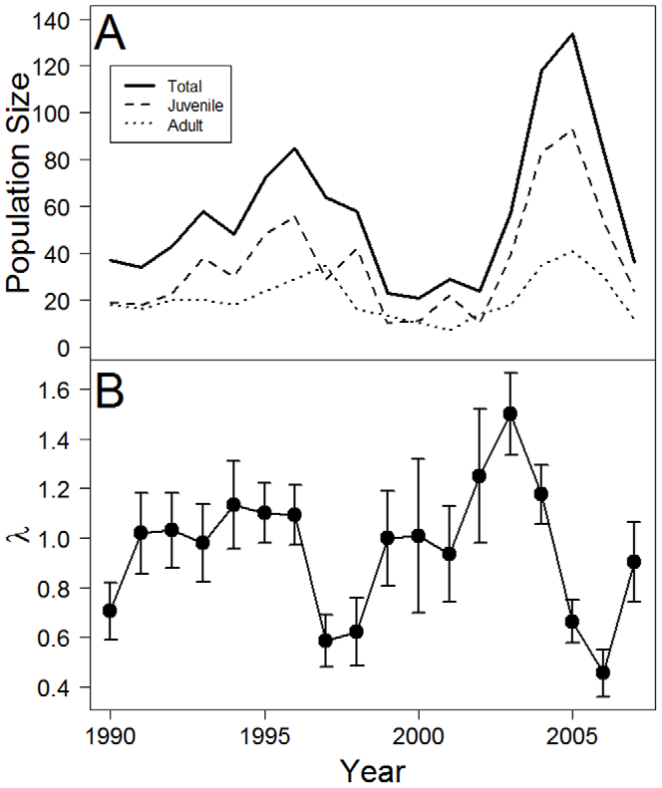
Annual population size and growth rates. (A) Total, juvenile, and adult population sizes and (B) deterministic population growth rate (*λ* ± SE) for a golden mantled ground squirrel population at the Rocky Mountain Biological Laboratory, Crested Butte, Colorado, for each year of the study.

The golden-mantled ground squirrel (*Callospermophilus lateralis*; formerly, *Spermophilus lateralis*; hereafter, GMGS) [18] is a hibernating species that is widely distributed in western North America, including subalpine habitats in the Rocky Mountains. We studied a population of GMGS at the Rocky Mountain Biological Laboratory in Colorado, USA, where climate change has been shown to influence life history and population dynamics of several species [15], [19]. Since the study began in 1990, our GMGS population has exhibited substantial fluctuations in size (Figure 1A) [20]. The availability of these long-term (1990–2008) demographic data allowed us to estimate annual vital demographic rates (survival, breeding probability, and litter size) and to test for the effects of population density as well as climatic variables on vital demographic rates. Using deterministic and stochastic demographic analyses, our goal was to investigate how density-dependent processes interacted with environmental stochasticity to influence dynamics and persistence of the GMGS population. First, we calculated overall and yearly deterministic population growth rates, and estimated potential and actual contributions of demographic vital rates to temporal changes in population growth rates. Second, we used stochastic demographic methods to calculate the stochastic population growth rate (*λ_s_*), and its elasticity to changes in the mean and variance of vital rates. Finally, we examined how density dependence and environmental stochasticity influenced population persistence parameters (probability of (quasi-) extinction and distribution of extinction times) when the influence of demographic stochasticity and immigration was or was not considered.

## Methods

### Study area and species

We conducted our research at the Rocky Mountain Biological Laboratory (RMBL) near Crested Butte, Colorado (38°58′N, 106°59′W, elevation 2890 m), USA, on a 13-ha subalpine meadow. The study area was interspersed with willow (*Salix sp.*) and aspen (*Populus tremuloides*) stands. The meadow was bordered by the East River to the west and Copper Creek to the south, which formed barriers to dispersal, and by aspen woodlands to the north and east that were uninhabited by GMGS.

The golden-mantled ground squirrel is an asocial and diurnal species that occurs at a broad range of elevations (∼1000–4000 m above sea level). It prefers open habitats such as mountain meadows and rocky slopes that are adjacent to grasslands [21], [22], [23], and such habitats were patchily distributed in our study area. The nearest localities supporting other GMGS populations were 250 m to the east and 300 m to the north. Dispersal in this species typically involve movements of <250 m but can exceed 1000 m [24]. The GMGS survives long winters, and therefore food shortage, via hibernation. Both altitude and amount of snowfall influence when squirrels commence and end their hibernation period [21], [22]. In our study area adult GMGS usually emerge from hibernation around the time of snow-melt (mid-May to early June). The breeding season closely follows emergence, and pups emerge from natal burrows late June to mid-July. At the end of summer (late August to early September) the squirrels enter hibernation.

At RMBL, GMGS primarily forage on herbaceous vegetation (forbs and grasses). Snow-melt greatly influences the growth of these green, leafy plants and hence impacts food availability for squirrels. Soon after emerging from hibernation, the squirrels begin gaining mass, rapidly storing fat to improve their chances of survival the next winter and to sustain gestation the next spring [25].

### Field methods

GMGS were live-trapped and observed for 19 successive years (1990–2008) during the active season (May to late August). The annual census (marking the entire resident population) took place from late May to early June. Pups were trapped and marked between late June and mid-July as litters emerged from their natal burrows. Squirrels were trapped also in late July and late August, in order to record their body masses as they were building fat reserves for hibernation. Throughout the summer, animals were observed daily and trapped opportunistically to capture and mark all new immigrants and refresh marks on residents [20], [26].

Squirrels were captured in single-door Tomahawk live-traps (12.7×12.7×40.6 cm) baited with a mixture of sunflower seeds and peanut butter. Once captured, squirrels were identified via numbered metal tags in each ear and were distinctly dye-marked with fur dye for visual recognition. Sex, mass and female reproductive condition were recorded, and new individuals received ear tags. Emerging pups were captured, dye-marked, and ear-tagged at first emergence from their natal burrow. Their mothers' ear tags were recorded as well as litter size.

A total of 831 squirrels was captured during the study period. Age was known for 704 squirrels because they were captured as juveniles when emerging from their natal burrows. We estimated age based on mass for immigrants, whose exact ages were unknown. Field methods followed protocols approved by the Animal Care and Use Committee at the University of California, Davis, and met guidelines of the American Society of Mammalogists [27].

### Matrix population model

We constructed and analyzed age-structured matrix population models, focusing on the female segment of the population because it was not possible to estimate reproductive parameters for males. Age of first reproduction was 1 year because many female squirrels reproduce as yearlings. Age of last reproduction was 6 years; of 326 known-age female squirrels, only one survived >6 yrs. The population projection matrix was of the following form:
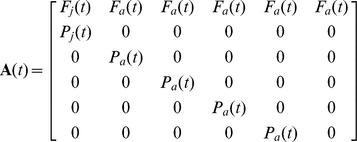
where (*t*) indicates time-specificity; *P_j_(t)* and *P_a_(t)* denote survival of juveniles and adults, respectively. The fertility rates for yearlings (i.e., age class 1) and adults (*F_j_(t)* and *F_a_(t)*, respectively) were calculated using post-breeding census methods as [28]: 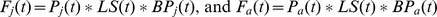, where *LS* is litter size and *BP_j_* and *BP_a_* denote breeding probability (i.e., probability of successful reproduction) of yearling and adult squirrels, respectively. Primary sex ratio was assumed to be 1∶1, as is typical of most ground squirrels [29].

### Deterministic demographic analysis

We constructed and analyzed overall and year-specific deterministic matrix models. For the overall or time-invariant model, a projection matrix **A** was constructed using age-specific estimates of vital rates based on capture-mark-recapture and reproductive data collected during the entire study period (1990–2008). For the year-specific models, a separate population projection matrix **A**(*t*) was compiled for each year of the study using age- and year-specific vital rate estimates; thus, we had 18 year-specific projection matrices. We calculated the overall and year-specific population growth rates as the dominant eigenvalues of the overall or year-specific population projection matrices, respectively. Elasticity of deterministic population growth rates to matrix entries and lower-level vital rates, net reproductive rate (*R_0_*), and generation time (*A*) were calculated using methods described by Caswell [28]. Finally, we used life-table response experiment (LTRE) analysis to decompose year-to-year changes in population growth rate into contributions from changes in matrix entries or underlying vital rates [28], [30] as:
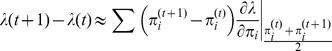
where *λ*(*t*+1) and *λ*(*t*) are growth rates in year *t*+1 and year *t*, respectively; *π_i_* is a matrix entry or a lower-level vital rate [31]. The term 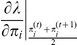 indicates that sensitivities were evaluated at the midpoint between values of *π_i_* in the 2 years being compared.

Overall estimates of demographic variables for the entire study period are presented in Table 1. Estimates of demographic variables and numbers of immigrants for each year of study are given in Figure S1.

**Table 1:** Mean and standard error (SE) of vital rates, as well as sensitivity and elasticity of overall deterministic population growth rate (i.e., based on vital rates estimated for the entire study period) to changes in vital rates for a golden-mantled ground squirrel population in Gothic, Colorado.

Parameter	Mean	SE	Sensitivity	Elasticity
*P_j_*	0.310	0.024	1.125	0.404
*P_a_*	0.519	0.029	1.081	0.596
*LS*	4.793	0.141	0.079	0.404
*BP_j_*	0.313	0.047	0.300	0.100
*BP_a_*	0.816	0.037	0.350	0.304

Vital rates are: juvenile survival (*P_j_*), adult survival (*P_a_*), litter size (*LS*), and breeding probability for yearlings (*BP_j_*) and older females (*BP_a_*).

### Stochastic demographic analysis

As noted previously, we compiled a population projection matrix **A**(*t*) for each year of the study using year-specific estimates of vital rates. We assumed independent and identically distributed (*iid*) environment such that vital rates observed in each of the 18 years of study were equally likely to occur. We used a simulation-based approach (50,000 time steps) to estimate the stochastic population growth rate and stochastic elasticities [28], [32], [33]. The stochastic population growth rate 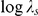 was calculated as: 
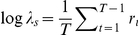
 where *r_t_* = log(*n*(*t*+1)/*n*(*t*)) is a one-step population growth rate (all logs are base *e*) [28], [32]. We estimated variance of log 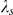 (σ^2^) using the log-normal approximation [28]. The approximate 95% CI of 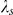 was then calculated as: 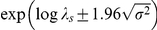
_._ We estimated elasticity of 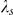 to matrix entries as [33]:
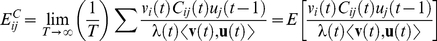
where **u**(t) and **v**(t) vectors refer to stochastic population structure and reproductive value at time *t*, *λ(t)* one time step population growth rate, and the term 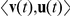 is the scalar product of vectors **v**(t) and **u**(t). We calculated three types of stochastic elasticities [33]. First, the overall stochastic elasticities 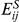 were calculated by setting 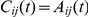 for every year *t*; elasticities of *λ_s_* to the mean of matrix elements 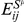 and variance of the matrix entries 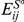 were obtained by setting 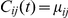
_,_ and 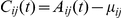
_,_ 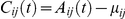, respectively [33]. Elasticities of *λ_s_* to lower-level vital rates were calculated using methods described by Caswell [34].

Influence of environmental and demographic stochasticity, density dependence, and immigration on population persistence.

We used a simulation-based approach to population viability analysis (PVA) using methods similar to those described in Morris and Doak [35]. We estimated population persistence parameters (probability of extinction/quasi-extinction and time to extinction) under a variety of scenarios, depending on whether and how the effects of environmental and demographic stochasticities, density dependence and immigration were modeled.

We used two approaches for incorporating environmental stochasticity in our simulations. In the first approach, we used yearly estimates of vital rates as described previously; simulations were conducted under the assumption that vital rates observed in each year of the study were equally likely to occur (hereafter, ES: year). The second approach was based on our earlier findings that average rainfall in June and July affected age-specific survival directly, and age-specific breeding probability with a 1-yr time lag (hereafter, ES: rainfall) [20]. The functional relationships between rainfall and age-specific survival rates were:
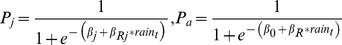
where *β*_0_ and *β_j_* are the intercept terms for adult and juvenile survival, respectively; *β_R_* and *β_Rj_* are slope parameters relating rainfall to adult and juvenile survival, respectively, and *rain_t_* is the mean June–July rainfall for year *t*.

The functional relationships between rainfall and age-specific breeding probability were:
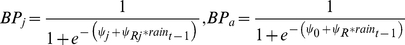
where *ψ*_0_ and *ψ_j_* are intercept terms for breeding probability of adult and juvenile females; *ψ_R_* and *ψ_Rj_* are slope parameters relating average June–July rainfall to breeding probability of adult and juvenile female squirrels, respectively.

We modeled demographic stochasticity (DS) using a sampling approach [28]. At each time step *t*, the number of survivors for all age classes *i* (*i*>1) was sampled from a binomial distribution with parameter *p* = age-specific survival probability, and *n* = number of females at *t*−1 in age class *i*−1. Likewise, the number of females that reproduced in year *t* was sampled from a binomial distribution with parameter *p* = age-specific breeding probability, and *n* = number of females in class *i*−1 surviving from year *t−1* to year *t*. We sampled the number of offspring produced by each female that reproduced from a zero-truncated Poisson distribution, with the Poisson parameter *μ* = mean litter size (adjusted to account for the zero-truncation). The total number of offspring produced by females of a given age class was then calculated as the sum of offspring produced by all females in that age class. The number of female offspring was sampled from a binomial distribution with parameter *n* = total number of offspring, and *p* = primary sex ratio (0.5). The number of individuals in age class 1 (juveniles) was the projected total number of female offspring produced by females of all age classes.

Density dependence has been suggested to be an important factor influencing dynamics and persistence of biological populations [4], [5], so we also evaluated population level effects of density dependence. In an earlier study, we found strong evidence for a delayed, negative effect of population density on age-specific survival [20]. The functional DD relationship for survival (*P_j_* and *P_a_*) is described by the following logistic regression equations:
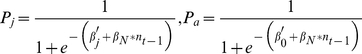
where *β*'s represent regression coefficients relating population size to age-specific survival (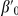: intercept term for adult survival, 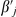: intercept term for juvenile survival, and 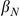: slope parameter relating the previous year's population density to survival). This density-dependent relationship was estimated using total population size (both sexes) and our population model was female-only, but the observed sex ratio did not vary much by year. Therefore, we divided corresponding female population size by the observed overall sex ratio (i.e., proportion of females) in the population (0.515) to extrapolate the approximate total population size from the number of females.

Some PVA scenarios included both ES and DD (see below). For ES: year, this was simulated by modeling survival as DD and all other parameters as varying by year. For ES: rainfall, we modeled BP as described previously and used the top-ranked model (based on Akaike Information Criterion corrected for small sample size [AIC_c_] [36]) incorporating ES and DD on vital rates to model survival [20]:
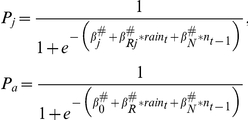
where *β^#^* indicates values of coefficients relating rainfall and population size to age-specific survival. Values of coefficients relating the effect of population density and average June–July rainfall on vital rates are given in Table S1.

We also modeled immigration as a stochastic process, using data on the number of immigrant females (≥1 yr) observed during annual censuses that took place soon after the emergence from hibernation (Figure S1). Dispersal in this species predominantly occurs late in the summer of birth [24], and most immigrants into our population were first recorded early the following year at age 1. Most immigrants were of unknown age; known aged squirrels were yearlings when they were first recorded in the annual census. To incorporate the influence of immigration on population dynamics and persistence, we randomly selected the number of yearling female immigrants each year from the 18 years of data (Figure S1). For the ES: year and immigration simulations, the number of immigrants was selected from the same year as the vital rates. Because the immigrants were counted before the birth pulse (i.e., pre-breeding census) but our population model was based on a post-breeding census formulation, it was necessary to include immigrants into the analysis in such a way that mortality of the immigrants was not included both explicitly (in the population model) and implicitly (mortality between the breeding season and when the immigrants were first counted). To do so, for simulations that included immigration and demographic stochasticity, we added the immigrants between the mortality and reproduction steps (assuming that immigrants had similar reproductive parameters as residents For simulations that included immigration but not demographic stochasticity, we projected the population as:
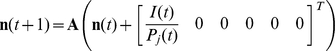
where *I(t)* is the number of immigrant yearling females at time *t*. This approach assumes that immigrant females had similar survival and reproductive rates as residents.

We projected population size for 50 years using the appropriate population projection matrix (or an equivalent algorithm when demographic stochasticity was considered) and an initial population vector **n**(0). The average number of females observed during our study (i.e., 30 females) was multiplied by the stable age distribution vector to obtain the initial population vector **n**(0). We projected the population size and calculated probabilities of (quasi-)extinction and distribution of extinction times under 24 scenarios. These scenarios included all combinations of environmental stochasticity (none, ES: year, and ES: rainfall); demographic stochasticity (none or all vital rates); density-dependence (none or density dependent survival); and immigration (none or random immigration of juvenile females).

We used MATLAB [37] for all calculations.

## Results

### Deterministic demographic analysis

The overall deterministic population growth rate (*λ*), calculated using vital rates estimated for the entire study period, suggested a population decline of 6% per year (*λ* = 0.94±SE 0.05) in the absence of immigration. However, 95% confidence interval included 1.0 (0.84–1.04), offering no statistical evidence for a population decline. Matrix entry elasticity analysis revealed that *λ* was proportionately most sensitive to changes in survival of juveniles (*P_j_*), followed by that in survival of 2-yr old females. Results of lower-level elasticity analysis showed that *λ* was proportionately most sensitive to changes in survival of adults (*P_a_*) (elasticity = 0.596), followed by that in *P_j_* and litter size (elasticity for both = 0.404), breeding probability for adults (elasticity = 0.304), and breeding probability for juveniles (elasticity = 0.10; Table 1). The net reproductive rate was 0.804 daughters per female per generation; generation time and life expectancy at emergence from the natal burrow was 2.74 and 1.62 years, respectively.

All vital rates varied substantially over time (Figure S1); coefficient of variation was 29.61%, 21.76%, 19.05%, 97.61%, and 18.28% for *P_j_*, *P_a_*, *LS*, *BP_y_* and *BP_a_*, respectively. Consequently, population growth rate also was highly variable over time, ranging from 0.45±0.09 in 2006 to 1.50±0.12 in 2003 (Figure 1B); it was >1 in 9 years, and <1 in 9 years. The pattern of elasticity was identical to that described above for the overall population in most years, except that in 2000 when elasticity of λ to survival of juveniles and litter size exceeded that to survival of adults, and elasticity of λ to breeding probability of juveniles exceeded that to breeding probability of adults. This was likely a consequence of the fact that all females one year of age or older successfully reproduced in 2000.

**Figure 2:**
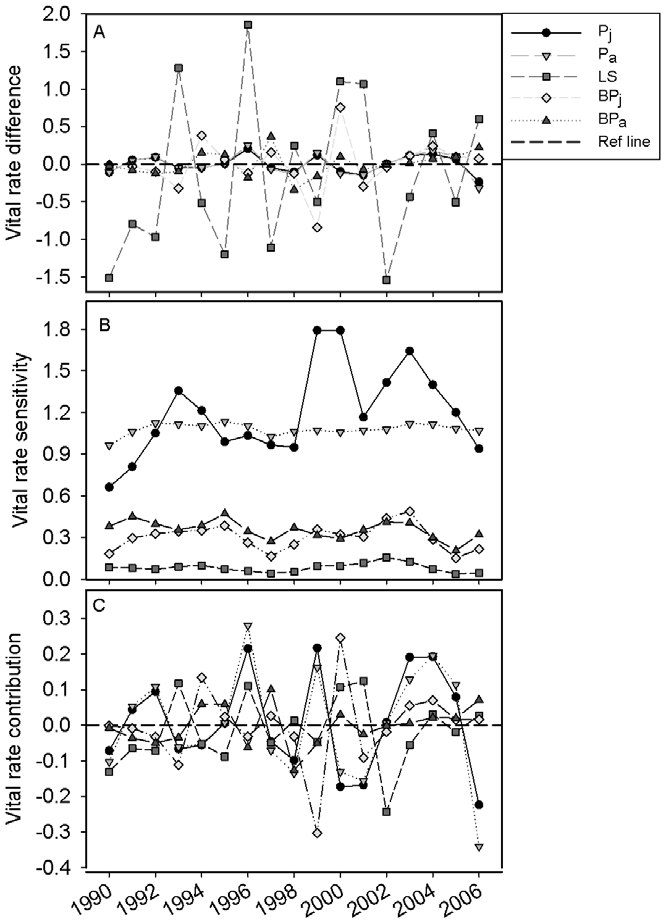
Contributions of vital rates to annual changes in population growth. Results of life table response experiment (LTRE) analysis: (a) differences in vital rates between consecutive years, (b) sensitivity of the deterministic population growth rate to changes in vital rates, evaluated at the midpoint between two successive years being compared, and (c) LTRE contribution of each vital rate to year-to-year changes in the deterministic population growth rate. Vital rates are: *P_j_* = juvenile survival, *P_a_* = adult survival, *LS* = litter size, *BP_j_* = breeding probability (i.e., probability of successful reproduction) for yearlings, and *BP_a_* = breeding probability for adults.

Contribution of vital rates to year-to-year changes in population growth rate also varied over time; this was as expected given that both vital rate values as well as sensitivity of λ to vital rates varied over time (Figure 2a–2c). The absolute value of LTRE contribution was in the following order (largest to smallest): *P_a_*, *P_j_*, *LS*, *BP_y_* and *BP_a_*. A Kruskal-Wallis test revealed that absolute values of LTRE contributions differed among vital rates (χ^2^ = 14.24, *P* = 0.007). However, the contribution of vital rates to year-year changes in *λ* varied over time (Figure 2c). Changes in *P_a_*, followed by that in LS made the largest contribution (absolute values) to year-to-year changes in *λ*, in 6 and 5 years, respectively. Two of the largest contributions of *BP_y_* occurred during 1999–2000 and 2000–2001 transitions, most likely because of the fact that all females 1 year of age or older successfully reproduced in that year; thus, changes in breeding probabilities from 1999 to 2000, and from 2000 to 2001 were rather substantial. The contribution of *P_j_* ranked 4^th^ in terms of frequency of largest contribution, although mean (absolute value) LTRE contribution of this variable was second only to that of *P_a_* (Figure 2c).

### Stochastic demographic analysis

**Figure 3:**
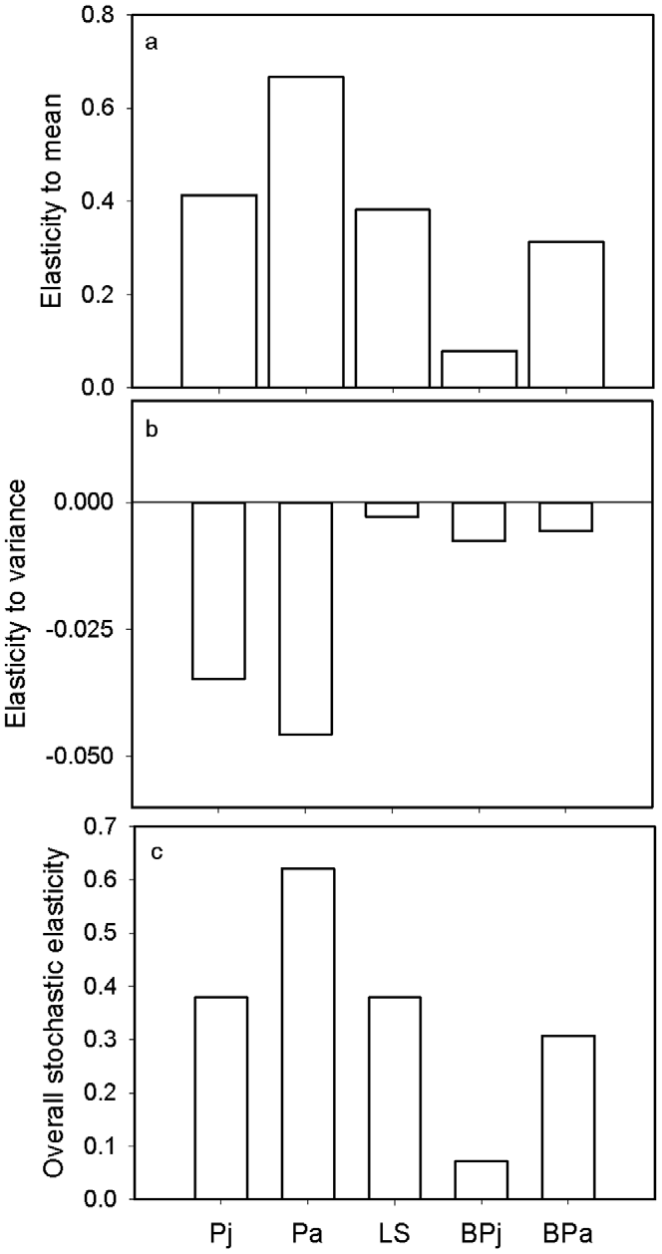
Proportional sensitivity of stochastic population growth rate to vital rates. Results of stochastic elasticity analysis: (a) elasticity of stochastic population growth rate (λ_s_) to changes in mean values of vital rates, (b) elasticity of *λ_s_* to changes in variance of vital rates, and (c) overall stochastic elasticities. Vital rates are: *P_j_* = juvenile survival, *P_a_* = adult survival, *LS* = litter size, *BP_j_* = breeding probability (i.e., probability of successful reproduction) for yearlings, and *BP_a_* = breeding probability for adults.

The stochastic population growth rate *λ_s_* was 0.92 (95% CI: 0.52–1.60); this value was less than, but statistically indistinguishable from, the overall deterministic population growth rate calculated from pooled estimates of vital rates (*λ_overall_* = 0.94; 95% CI: 0.84–1.04) or that based on the mean matrix (*λ_mean_* = 0.95), as is typical. Stochastic vital rate elasticities revealed a pattern similar to deterministic elasticities, and showed that *λ_s_* was proportionately most sensitive to changes in the mean and variance of *P_a_*, followed by that of *P_j_* and *LS*. The elasticity of *λ_s_* to vital rate variances was negative, indicating that an increase in vital rate variance would reduce stochastic population growth rate (Figure 3). The overall stochastic elasticities displayed essentially the same pattern.

The influence of environmental and demographic stochasticities, density-dependence and immigration on population dynamics and persistence.

**Figure 4:**
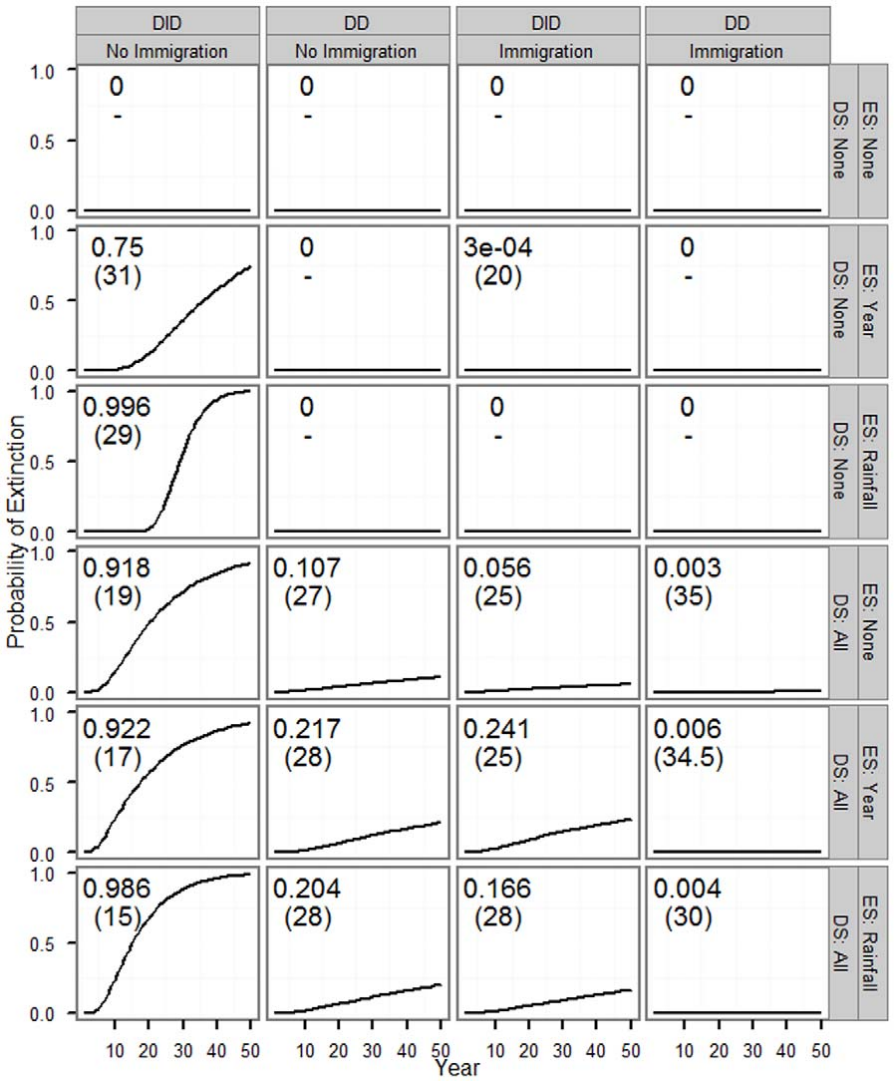
Probability of extinction by simulation scenario. The cumulative probability of extinction during a 50-yr period (i.e., probability that the population falls below one female) across 24 simulation scenarios depending on whether and how density dependence, immigration, demographic stochasticity, and environmental stochasticity were modeled. Scenarios are as follows: density-dependence - DID (density-independent vital rates) and DD (density-dependent survival); immigration - immigration ignored (No Immigration), and immigration included (Immigration); environmental stochasticity - environmental stochasticity ignored (ES: None), environmental stochasticity modeled with annual estimates of vital rates (ES: Year), and environmental stochasticity modeled with effects of average June–July rainfall on vital rates (ES: Rainfall); and demographic stochasticity - demographic stochasticity ignored (DS: None), and demographic stochasticity considered in all vital rates (DS: All). Cumulative probability of extinction for each scenario based on 10,000 simulations is represented by solid line. Probability of extinction within 50 years and median extinction time for each scenario are presented in large text within each figure panel. All scenarios started with 30 females, distributed to age classes according to stable age distribution for the overall population.

**Figure 5:**
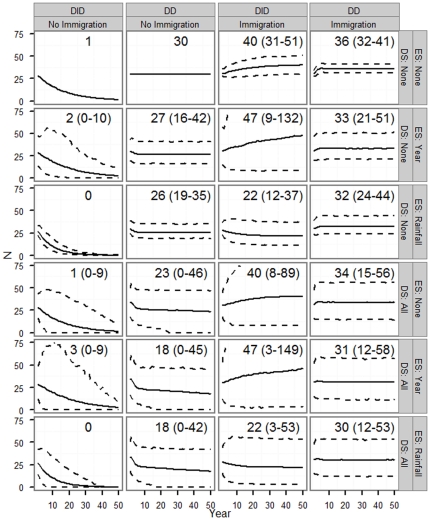
Population sizes by simulation scenario. Projected mean population size (solid lines) and 90^th^ percentile (dashed lines) of projected population sizes based on 10,000 simulations across 24 scenarios depending on whether and how density dependence, immigration, demographic stochasticity, and environmental stochasticity were modeled. Mean projected population size and 90th percentiles in year 50 (rounded to the nearest integer) also are given for each scenario within the figure panels. See Figure 4 for the description of scenarios and other simulation details.

The probability of extinction (the probability that the projected population size falls below 1 female) as well as median time to extinction varied widely across 24 scenarios depending on whether and how density dependence, immigration, demographic stochasticity, and environmental stochasticity were modeled (Figure 4). When density dependence and immigration were ignored but some form of stochasticity was included, probability of extinction (PE) within 50 years was generally high (≥0.75, mostly very close to 1). In contrast, the probability of extinction was at or near zero when both density dependence and immigration were considered; the probability of extinction remained close to zero even when demographic and/or environmental stochasticity were considered. Probability of extinction and median time to extinction were intermediate (0.05<PE<0.25) when either density dependence or immigration (but not both) and demographic stochasticity were considered. Including additional source of stochasticity generally increased probability of extinction. As expected for small populations, demographic stochasticity generally had a greater impact on population persistence than environmental stochasticity. For the scenario with no stochasticity, density dependence or immigration the population size at year 50 was just above 1, with PE = 0 (Figure 5); when environmental and/or demographic stochasticity was included, however, PE increased substantially. For scenarios with non-zero extinction probabilities, median time to extinction varied between 15 and 35 years, with generally higher values of median time to extinction for scenarios with density dependence and immigration (Figure 4).

Mean projected population sizes varied across scenarios (Figure 5). Scenarios with DID and no immigration had very low or zero average population sizes by year 50, in part because extinct populations (*N*<1) were included in the calculation of averages. Scenarios with density dependence, no immigration, and no DS had mean population sizes of 26–30 females by year 50; when demographic stochasticity was added, this dropped to 18–23 females. Scenarios with DD and immigration had mean population sizes of 30–36 females by year 50; demographic or environmental stochasticity generally had little effect. Projected mean population sizes were generally higher for scenarios that considered immigration (Figure 5).

We also calculated probability of quasi-extinction (i.e., probabilities that the population falls below a critical population size, *N_crit_*) for *N_crit_* = 5 and *N_crit_* = 10 females. Quasi-extinction probabilities were generally higher than extinction probabilities, and increased further as the critical population size increased (Figure S2, Figure S3). Probabilities of quasi-extinction were particularly high for scenarios with DS, especially for *N_crit_* = 10. Median time to quasi-extinction was lower than median time to extinction, and varied between 11 and 30 years for *N_crit_* = 5 and 2 and 30 for *N_crit_* = 10.

## Discussion

Virtually all natural populations experience stochastic environmental variations which can influence demographic variables, and population dynamics and persistence [12], [33], [38]. Whereas environmental stochasticity tends to destabilize population dynamics [39], [40], density-dependent mechanisms tend to have stabilizing effects and eventually lead to population regulation [4], [6], [8], [41], [42]. When population sizes are small, demographic stochasticity can also be an important influence on population persistence [43], and immigration can help reduce extinction risks in open populations. Understanding how these factors interact to affect population dynamics and persistence is especially important for species that occupy habitats sensitive to climate change. This is because global climate change can potentially accentuate the destabilizing effect of environmental stochasticity, and thus can profoundly influence population dynamics and persistence [16], [17], [44], [45].

Our goal was to understand factors and processes influencing dynamics and persistence of a golden-mantled ground squirrel (GMGS) population inhabiting a montane habitat where the changing climate is affecting life history and population dynamics of several species [15], [19]. The total size of our study population ranged from 24 squirrels in 1999 and 2000 to 140 in 2005, almost a 6-fold difference (Figure 1A) [20]. Likewise, the population growth rate varied over time (Figure 1B) reflecting substantial temporal environmental variation, a pattern also observed in other sympatric hibernating squirrels [46], [47]. Deterministic prospective and retrospective perturbation analyses revealed that changes in survival of juvenile and adult females were primarily responsible for observed annual variation in population growth rate, although reproductive parameters also were important especially when they experienced large changes. The stochastic growth rate *λ_s_* was lower than the deterministic growth rate of the mean matrix. Stochastic elasticity patterns were similar to the pattern of deterministic elasticities, and revealed that *λ_s_* was proportionately most sensitive to changes in mean and variance of adult and juvenile survival rates.

In addition to the broad population fluctuations, we have witnessed population lows with as few as five adult female squirrels in 1999 and 2001 [20]. During the summer of 2001, the adult female population size dipped to three individuals because two females disappeared from the study site after the annual census, most likely due to predation. Yet, the population proved resilient as it re-bounded and has not yet gone extinct. What are the factors and processes that allowed the relatively small population of GMGS to persist? To address this question, we performed population viability analysis under 24 scenarios, depending on whether and how density dependence, environmental stochasticity, demographic stochasticity and immigration were modeled. When density dependence and immigration were ignored but stochasticity was considered, the population had a very high (>0.75; mostly close to 1.0) probability of extinction, and the most likely time to extinction was as early as 15 years. The probability of extinction declined substantially and somewhat similarly when either immigration or density dependence was considered; when the effects of immigration and density dependence were considered simultaneously, the probability of extinction practically declined to zero (Figure 4). Finally, the influence of demographic stochasticity was strong, as predicted by theory for small populations [28], [48]. These results conclusively demonstrate that stabilizing effects of density dependence and rescue effects of immigration counteracted destabilizing stochastic influences on our study populations, and that in the absence of density-dependent regulation and immigration, small populations are under substantial extinction risk.

Both the overall deterministic and stochastic population growth rates were proportionately most sensitive to changes in survival of adult and juvenile survival – the two vital rates that have also been shown to be density-dependent [20]. Based on these results, we conclude that density-dependent survival and rescue effect of immigration have allowed our study population to persist in the face of stochastic influences. Our results add to the body of evidence suggesting that many biological populations are likely regulated by synergistic effects of deterministic (e.g., density dependence) and stochastic (e.g., environmental and demographic stochasticity) factors [3], [7], [9].

In the last 18 years, the total female population size (including juveniles) has never dropped below 10 [20]. This is consistent with our PVA results where the probability of the population dropping below 10 females within 18 years was only 21–24% for scenarios that considered demographic and environmental stochasticity, immigration and density-dependence (Figures S2 and S3). Over the longer time span of 50 years, however, this probability increases to ≥50%.

Several authors have pointed out that environmental stochasticity based on annual estimates of vital rates may be biased high due to confounding of sampling error and process variance [49], [50], [51]. Conversely, estimates of environmental stochasticity based on environmental factors may be biased low due to effects of unmeasured environmental covariates. We tested for the effects of environmental stochasticity estimated based on annual estimates of vital rates (ES: Year) and those based on the effects of summer rainfall on vital rates (ES: Rainfall), and evaluated how these alternative approaches to quantifying environmental stochasticity affected extinction parameters. The probability of extinction and median time to extinction obtained from the two approaches were often similar. When probability of extinction estimated based on the two approaches to ES differed, the estimate from ES: Rainfall was generally closer to that obtained from analyses that ignored environmental stochasticity than that based on ES: Year; the only exception was the scenarios that ignored DD and immigration; Figure 4). It seems likely that the actual effect of environmental stochasticity on the dynamics and persistence of our study population lies between the two approaches considered here.

Causes and population dynamic consequences of immigration (and emigration) have been an active area of research in ecology [52], [53]. Although immigration is thought to be necessary for metapopulation persistence [54], its role in local population dynamics is still debated [55], [56]. In some species of small mammals, the role of immigration in local population dynamics is considered to be minor (e.g. [57], [58], [59]). Our results suggest that immigration is an important factor contributing to dynamics and persistence of our study population. Without immigration, our study population would have faced a high likelihood of extinction during a population bottleneck that occurred from 1999–2002; the population size during that period was reduced to ≤14 adults (Figure 1A). An influx of immigrants in 2002 and 2004 most likely prevented population extinction and loss of genetic variation and inbreeding [26]. Despite a fairly low rate of immigration to our population (mean = 1.17 females/year), our simulation results suggest that immigration dramatically reduces the probability of extinction (from >20% within 50 years to l<1%, when DD, DS, and ES are considered simultaneously). Although our study focused on a single local population, it is clear that this population exists as part of larger metapopulation with demographic and genetic connections among local populations [26]. Exchange of individuals among local populations was clearly important in population persistence as well as maintenance of genetic diversity.

The influence of environmental stochasticity is likely to be exacerbated by the effect of the predicted global climate change. Indeed, the climate is changing in our study site, and the changing climate has been shown to influence the life history of several species [15], [19]. One possible mechanism by which climate change could influence our study population is via changes in summer rainfall patterns. The average rainfall during summer months (June–July) has been shown to influence both survival and probability of successful reproduction in our study population [20], and this can influence both probability of extinction and median time to extinction (Figs. 4–5). Similar population-level effects of climate change have been predicted for several species [11], [16], [17].

Despite substantial population fluctuations, our study population has bounced back from low numbers and persisted to date. The regulatory effect of density dependence and the rescue effect of immigration will likely allow this population to persist for years to come. Nonetheless, the GMGS population is likely to face substantial extinction risk, especially if regulatory influences are weakened or if habitat or climate change reduces the rate of immigration into the study population such as that observed in the endangered Idaho ground squirrel (*Urocitellus brunneus*) [60]. Stochastic processes such as environmental and demographic stochasticity as well as increases in the mean and variability of summer precipitation would undoubtedly increase vulnerability of our study population to extinction. The earth's climate is changing, and the changing climate will undoubtedly affect the distribution, abundance, and persistence of populations [14], [61]. A daunting future challenge for ecology is to be able to understand and predict how these changes would influence biological populations and communities [17], [42], [45], [62].

## Supporting Information

Figure S1 Annual mean (±SE) values of vital rates and annual number of immigrants.
(DOC)

Table S1 Regression coefficients relating summer rainfall and population density to age-specific survival and breeding probabilities.
(DOC)

Figure S2 Cumulative probabilities of quasi-extinction (i.e., the probability that the simulated population falls below 5 females) across simulation scenarios.
(DOC)

Figure S3 Cumulative probabilities of quasi-extinction ((i.e., the probability that the simulated population falls below 10 females) across simulation scenarios.
(DOC)
