# Supplementary material for: Stochastic Population Dynamics of a Montane Ground-Dwelling Squirrel
Source: PLoS One. 2012 Mar 27;7(3):e34379. doi: 10.1371/journal.pone.0034379 (PMC3313969; doi:10.1371/journal.pone.0034379)
Supplement: Figure S1 — Annual mean (±SE) values of vital rates and annual number of immigrants. (DOC) [file pone.0034379.s001.doc]

**Figure S1**

Mean (±SE) values of: (a) juvenile survival (*P_j_*), adult survival (*P_a_*), breeding probability for yearlings (*BP_j_*) and older females (*BP_a_*); and (b) litter size (*LS*) and number of immigrants for a golden-mantled ground squirrel population in Gothic, CO for each year of the study.
