# Supplementary material for: Stochastic Population Dynamics of a Montane Ground-Dwelling Squirrel
Source: PLoS One. 2012 Mar 27;7(3):e34379. doi: 10.1371/journal.pone.0034379 (PMC3313969; doi:10.1371/journal.pone.0034379)
Supplement: Table S1 — Regression coefficients relating summer rainfall and population density to age-specific survival and breeding probabilities. (DOC) [file pone.0034379.s002.doc]

**Table S1**

Definition and values of regression coefficients relating summer rainfall and population density to age-specific survival and breeding probabilities. See Methods section in the main text for detailed description of these relationships and for equations.

| Coefficient | Definition | Value |
| --- | --- | --- |
| *β*_0_ | Adult intercept for rainfall-dependent survival | -0.246 |
| *β_j_* | Juvenile intercept for rainfall-dependent survival | 0.098 |
| *β_R_* | Adult current year rainfall effect for rainfall-dependent survival | 0.003 |
| *β_Rj_* | Juvenile current year rainfall effect for rainfall-dependent survival | -0.008 |
| *β*_0_' | Adult intercept for density-dependent survival | 0.807 |
| *β_j_'* | Juvenile intercept for density-dependent survival | -0.065 |
| *β_N_* | Effect of previous population size for density-dependent survival | -0.011 |
| *β*_0_*^#^* | Adult intercept for rainfall- and density-dependent survival | 0.362 |
| *β_j_^#^* | Juvenile intercept for rainfall- and density-dependent survival | 0.751 |
| *β_R_^#^* | Adult rainfall effect for rainfall- and density-dependent survival | 0.004 |
| *β_Rj_*^#^ | Juvenile rainfall effect for rainfall- and density-dependent survival | -0.007 |
| *β_N_^#^* | Effect of population size for rainfall- and density-dependent survival | -0.011 |
| *ψ*_0_ | Adult intercept for rainfall-dependent breeding probability | 2.703 |
| *ψ_j_* | Yearling intercept for rainfall-dependent breeding probability | 2.030 |
| *ψ_R_* | Adult rainfall effect for rainfall-dependent breeding probability | -0.010 |
| *ψ_Rj_* | Yearling rainfall effect for rainfall-dependent breeding probability | -0.033 |
