# Supplementary material for: Stochastic Population Dynamics of a Montane Ground-Dwelling Squirrel
Source: PLoS One. 2012 Mar 27;7(3):e34379. doi: 10.1371/journal.pone.0034379 (PMC3313969; doi:10.1371/journal.pone.0034379)
Supplement: Figure S2 — Cumulative probabilities of quasi-extinction (i.e., the probability that the simulated population falls below 5 females) across simulation scenarios. (DOC) [file pone.0034379.s003.doc]

**Figure S2**

The cumulative probability of quasi-extinction for critical population size *N_crit_* = 5 females during 50-yr period (i.e., probability that the population falls below 5 females) across 24 simulation scenarios depending on whether and how density dependence, immigration, demographic stochasticity and environmental stochasticity were modeled. Cumulative probability of quasi-extinction for each scenario based on 10,000 simulations is represented by solid line.  Probability of quasi-extinction within 50 years and median extinction time for each scenario are presented in large text within each figure panel.  See Fig. 4 for the description of scenarios and other simulation details.
