# Supplementary material for: Stochastic Population Dynamics of a Montane Ground-Dwelling Squirrel
Source: PLoS One. 2012 Mar 27;7(3):e34379. doi: 10.1371/journal.pone.0034379 (PMC3313969; doi:10.1371/journal.pone.0034379)
Supplement: Figure S3 — Cumulative probabilities of quasi-extinction ((i.e., the probability that the simulated population falls below 10 females) across simulation scenarios. (DOC) [file pone.0034379.s004.doc]

**Figure S3**

The cumulative probability of quasi-extinction for critical population size *N_crit_* = 10 females during 50-yr period (i.e., probability that the population falls below 10 females) across 24 simulation scenarios depending on whether and how density dependence, immigration, demographic stochasticity and environmental stochasticity were modeled. Cumulative probability of quasi-extinction for each scenario based on 10,000 simulations is represented by solid line.  Probability of quasi-extinction within 50 years and median extinction time for each scenario are presented in large text within each figure panel.  See Fig. 4 for the description of scenarios and other simulation details.
